# Damage Mechanism and Modeling of CFRP Laminates Impacted by Single Waterjets: Effect of the Impact Direction

**DOI:** 10.3390/ma18153495

**Published:** 2025-07-25

**Authors:** Naidan Hou, Yulong Li, Ping Liu

**Affiliations:** 1 Institute of Systems Engineering, China Academy of Engineering Physics, Mianyang 621999, China; liuping_caep@163.com; 2 School of Aeronautics, Northwestern Polytechnical University, Xi’an 710072, China; 3 Shaanxi Key Laboratory of Impact Dynamics and Engineering Application, Xi’an 710072, China; liyulong@nwpu.edu.cn; 4 School of Civil Aviation, Northwestern Polytechnical University, Suzhou 215400, China

**Keywords:** waterjet impact, impact angle, fiber orientation, damage modeling, CFRP

## Abstract

In engineering practice, liquid droplet impingement typically occurs at an oblique angle relative to the target surface, yet the influence of impact orientation on damage outcomes remains contentious and exhibits target-material dependency. In this paper, a typical single-waterjet-generating technique is applied to liquid impact tests on a unidirectional carbon fiber-reinforced polymer (CFRP) laminate, with special focus on the effects of the impingement angle and the fiber orientation. Finite-element simulation is employed to help reveal the failure mechanism of oblique impacts. The results show that, in most cases, the damage caused by a 15° oblique impact is slightly larger than that of a normal impact, while the increase amplitude varies with different impact speeds. Resin removal is more prone to occur when the projection of the waterjet velocity on the impact surface is perpendicular (marked as the fiber orientation PE) rather than parallel (marked as the fiber orientation PA) to the fiber direction of the top layer. A PE fiber orientation can lead to mass material peeling in comparison with PA, and the damage range is even much larger than for a normal impact. The underlying mechanism can be attributed to the increased lateral jet-particle velocity and resultant shear stress along the impact projection direction. The distinct damage modes observed on the CFRP laminate with the different fiber orientations PE and PA originate from the asymmetric tensile properties in the longitudinal/transverse directions of laminates coupled with dissimilar fiber–matrix interfacial characteristics. A theoretical model for the surface damage area under a single-jet impact was established through experimental data fitting based on a modified water-hammer pressure contact-radius formulation. The model quantitatively characterizes the influence of critical parameters, including the jet velocity, diameter, and impact angle, on the central area of the surface failure ring.

## 1. Introduction

Erosion by liquid droplet impingement (LDI) is an important topic of interest in industrial fluid mechanics and material science. Such erosion has been observed in pipelines of nuclear and fossil power plants [1,2], in cracks initiated on gas-turbine blades [3,4], in rain erosion of the material of aircrafts [5,6], and in wind turbines operating under rainy conditions [7,8]. The single-waterdrop impact on a solid surface is a fundamental problem in this field, which has been investigated for decades and is generally understood [9,10,11]. In the early stage of impingement, the liquid behaves in a compressible manner and generates the so-called water-hammer pressure within the contact area, which can easily reach a magnitude higher than the yield strengths of most engineering materials, including metals, ceramics, and polymers. When the shock wave in the liquid exceeds the expansion of the contact periphery, the liquid outflow will eject laterally along the solid surface and finally become an incompressible steady flow with much lower Bernoulli static pressure [12].

Under typical operational conditions, raindrop impingement on material surfaces occurs at oblique angles due to the aircraft angle of attack or turbine-blade mounting configurations. The existing research generally indicates that the extent of impact-induced failures diminishes with increasing deviation from the perpendicular incidence. Hand et al. [13] established a theoretical framework for oblique droplet impacts, identifying the attenuation of water-hammer pressure through velocity vector decomposition (scaled by cos θ) and geometric reduction of the high-pressure contact area (proportional to cos^2^ θ). Hattori and Kakuichi [14] conducted spray-nozzle tests on different types of carbon steel and revealed that the maximum depth of erosion rate can be estimated using *V*cos^n^ θ, in which *V* represents the impact velocity and the exponent *n* is 1.2–2.5. On the contrary, experimental observations reveal material-specific anomalies. Gorham and Field [15] demonstrated through single-impact testing that peak surface erosion occurs at 10–25° off-normal angles under critical velocity thresholds, which may be blamed for the “enhanced lateral jetting velocity” in the downstream direction of the obliquely impacting surface. Matthewson and Gorham [16] documented the enhanced damage at low-angle impacts (5–15°) on glass-fiber composites compared with a normal incidence. Recent experimental investigations by Singh et al. [17] employing rotational erosion testing (30–70 m/s) on glass-fiber-reinforced polymer (GFRP) turbine-blade composites revealed a non-monotonic angular dependence of material degradation, with peak erosion rates occurring at a 45° oblique incidence rather than under normal impact conditions. Most explanations attribute these deviations to enhanced lateral jetting phenomena, although the stress magnitude and damage progression extent induced by this “strengthening effect” on material surfaces requires further investigation.

Previous investigations have demonstrated that the fiber orientation angle (defined as the angular deviation between the surface fiber direction and the impact vector) exerts significant influence on the erosion behavior of CFRP laminates [18]. Solid-particle erosion studies on CFRP composites reveal greater material removal under a parallel fiber orientation relative to a perpendicular alignment, which is attributable to an inferior transverse bending strength [19]. However, limited attention has been devoted to understanding fiber orientation effects on rain-erosion damage mechanisms in CFRP structures. Our preliminary investigations employing a pulsed-jet-generation apparatus (producing a continuous waterjet impact at 320 m/s with a 2 mm diameter) identified the following orientation-dependent damage asymmetries: matrix crack-propagation bias under a parallel fiber orientation and interlaminar delamination asymmetry in perpendicular configurations during oblique impacts [6]. Nevertheless, investigations remain inadequate regarding potential variations in damage characteristics or underlying mechanisms under rainfall conditions involving higher velocities (400–600 m/s) and larger droplet diameters (2–5 mm).

In this paper, a typical single-waterjet-generating technique is applied to liquid impact tests on a unidirectional (UD) CFRP laminate, with special focus on the effect of the impingement angle and the fiber orientation. Surface damage characteristics are examined by high-resolution optical microscopy, and internal delamination is detected by ultrasonic C-scan devices. Finite-element simulation is also employed to add a coherent perspective to the understanding of the impact procedure and failure mechanism. An enhanced damage model was formulated based on the water-hammer-pressure theory and calibrated using experimental data to quantitatively characterize the angular dependence of waterjet damage on composite materials. The objective of the present study is to reveal whether and how the impact angle and relative fiber orientation in UD composites affect their damage behavior during liquid impact.

## 2. Experiment Setup and Materials

**Figure 1:**
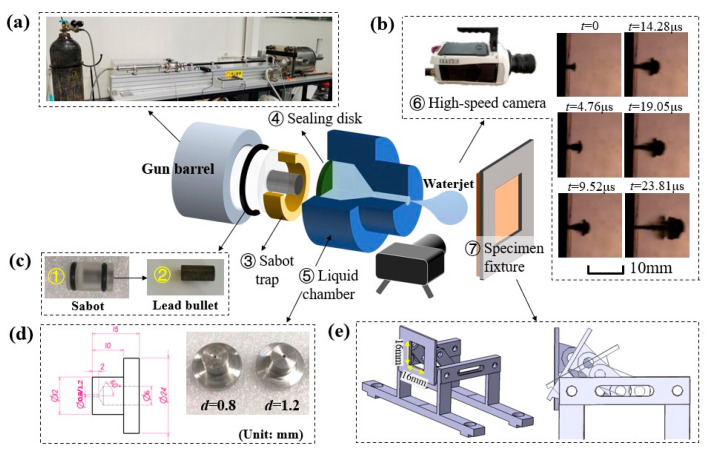
Experiment apparatus. (**a**) Air-gun barrel. (**b**) High-speed photography of the waterjet generation. (**c**) Bullet. (**d**) Liquid chamber. (**e**) Specimen fixture.

In this research study, the waterjet-generation system is designed following the operational framework of the single-impact waterjet apparatus (SIJA), as pioneered by Cavendish Laboratory. A detailed illustration of the experimental setup can be found in Figure 1, and its principle has been speculated on in [20,21]. The experimental setup consists of a cylindrical steel vessel equipped with a converging nozzle, which is filled with tap water and sealed using a neoprene diaphragm. Upon initiation of the test, a projectile is fired down the barrel, striking the diaphragm at a high velocity. This impact generates a transient pressure wave, which forces the water through the nozzle and produces a high-velocity liquid jet. The morphological changes of the moving waterjet are captured by high-speed photography, and hence the waterjet velocity can be calculated. The velocity of the waterjet can be controlled by varying the pressure of the driving gas, while the diameter of the waterjet is determined by selecting reservoirs with different nozzle diameters. In this study, waterjets with a speed between 350 m/s to 650 m/s and a diameter between 4 to 6 mm were generated for tests. A microscope with a magnification of 20~200× is used for observing and measuring the surface damage, and ultrasonic C-scan devices with a sound frequency of 5 MHz are utilized to examine the internal damage.

**Figure 2:**
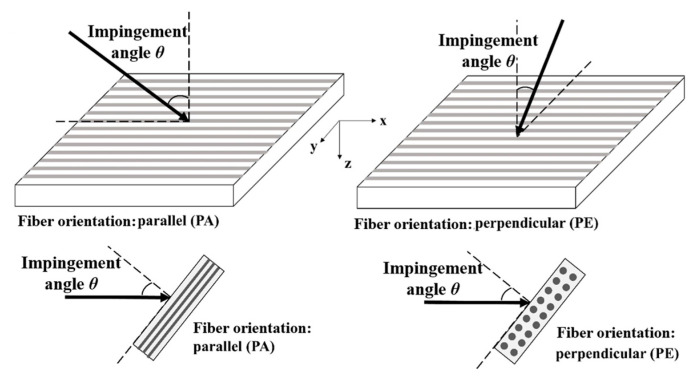
Definition of the impingement angle and the fiber orientation.

The carbon fiber-reinforced polymer (CFRP) laminates were fabricated using unidirectional T700/7901 prepreg (carbon fiber/epoxy composite) supplied by Guangwei Co. Ltd. (Shandong, China), with their mechanical properties documented in Table 1. The laminates were constructed by stacking 16 plies in a symmetric configuration of [0,90]_4s_; these were then vacuum-bagged and subjected to an autoclave curing at 120 °C for 90 min under a constant pressure of 3 bars in accordance with the process parameters recommended by the manufacturer. The final specimens were cut with a diamond wire saw into dimensions of 30 mm × 30 mm with a nominal thickness of about 2 mm. Each specimen was mounted onto a fixture that is flexible enough to adjust the inclination angle from 0° to 90°, as shown in Figure 1e. In this study, the impingement angle (θ) is defined as the inclination between the waterjet direction and the normal surface of the target. The fiber orientation is characterized by the alignment of the waterjet’s velocity projection relative to the top fiber layer’s axis—either perpendicular (PE) or parallel (PA), as illustrated in Figure 2. Four impact angles of θ = 0° (normal impact), 15°, 30°, and 45° were considered, and a total of 7 working conditions under two fiber orientations of PE and PA were labeled as 0°, 15°-PE, 15°-PA, 30°-PE, 30°-PA, 45°-PE, and 45°-PA.

## 3. Results and Discussion

### 3.1. Typical Damage Characterization

**Figure 3:**
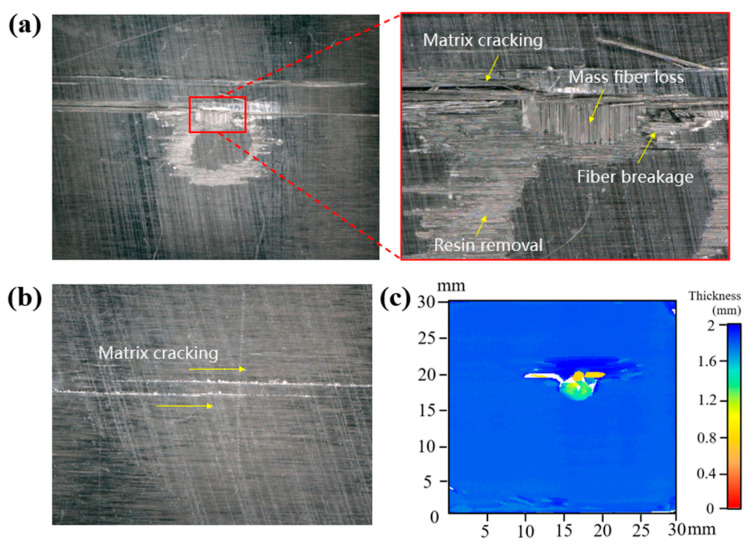
Typical damage patterns of the CFRP laminate impacted by a waterjet with a velocity of 590 m/s and a diameter of 4.8 mm. (**a**) The impacted surface damage, (**b**) back surface damage, and (**c**) C-scan result of the internal damage.

**Figure 4:**
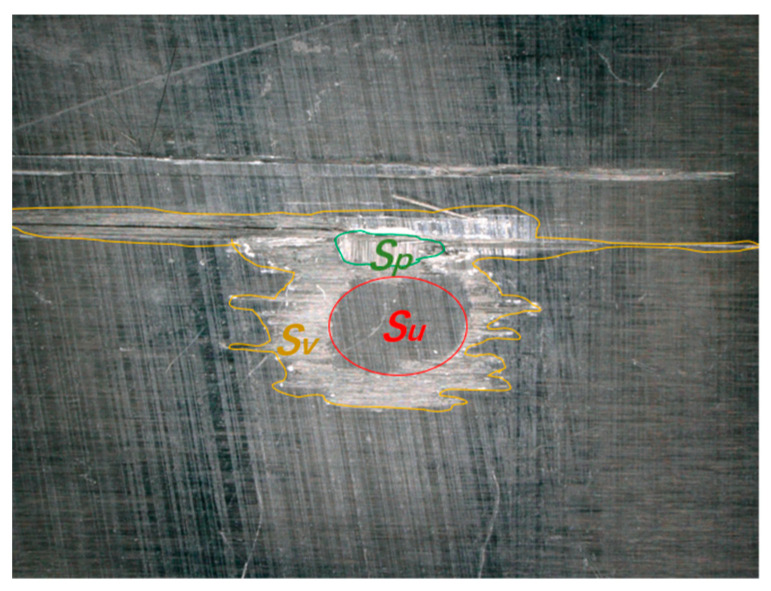
Definition of typical damage parameters.

Figure 3 shows the typical damage to the CFRP laminate after the normal impingement of a single waterjet with a velocity of 590 m/s and a diameter of 4.8 mm. There is an almost circular undamaged central area and a faded “failure ring” presented on the impacted surface damage shown in Figure 3a, with resin removal, matrix cracking, fiber breakage, and a small area of fiber loss. In addition, a series of matrix cracks parallel to the fiber direction was found on the back of the specimen, as shown in Figure 3b. The C-scan results of the same specimen is shown in Figure 3c in order to study the internal damage. It can be clearly observed that there is a spindle-shaped delamination area near the impact center that shows obvious asymmetry, with a longitudinal length of 10 mm and a transverse length of 4.5 mm. All the above damage characteristics of the CFRP laminate, as well as the underlying mechanisms for the normal impact of a single waterjet, have been analyzed in previous studies [20,22]. In the subsequent text, to quantify the extent of the damage, typical damage parameters of the CFRP sample surface after a single high-speed waterjet impact are defined as shown in Figure 4. The total area of surface damage visible under a microscope is defined as *S*_v_ (since the surface damage starts from matrix damage, this area can also be regarded as the area of surface matrix damage). The area of surface layer peeling (i.e., the fiber loss area) is defined as *S*_p_, and the area of the undamaged center of the surface failure ring is defined as *S*_u_.

### 3.2. Oblique Impact Results

#### 3.2.1. Effect of the Impingement Angle

**Figure 5:**
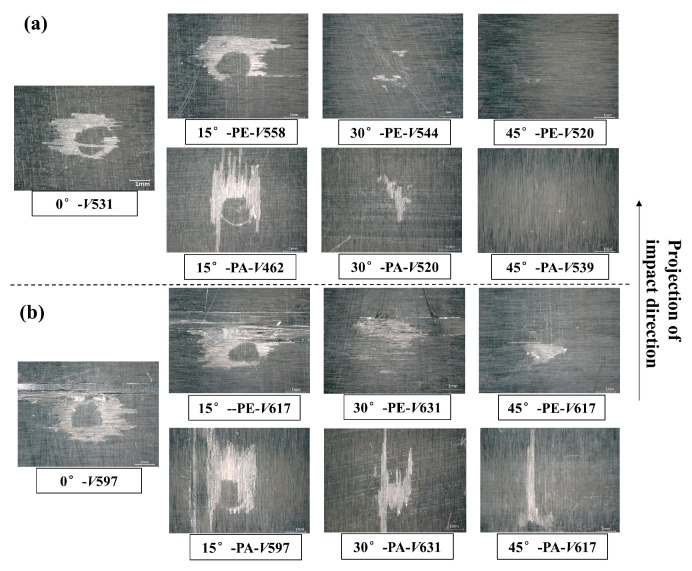
Typical damage morphology of CFRP laminates after the impact of single jets under seven different impact directions with two grades of waterjet velocity: (**a**) 520~544 m/s; (**b**) 597~631 m/s.

Figure 5 shows the typical damage morphology under the impact of single waterjets with an average diameter of 4.5 mm from the seven different impact directions defined in Section 2. Figure 5a shows the damage results, which are mainly caused by resin removal in the failure ring when the jet velocity is 520~544 m/s. When the impact angle increases from 0° to 15°, the damage degree does not change significantly, while the failure ring begins to show asymmetric characteristics, and the resin removal area is inclined along the impact projection direction. When increasing from 15° to 30° and 45°, the surface damage degree decreased significantly, and even the typical annular surface damage could not be identified. Comparing the results of the two fiber orientations under the same impact angle, it was found that there was no obvious difference between the PA and PE. As shown in Figure 5b, when the waterjet velocity increased to 597–631 m/s, more serious damage modes such as matrix cracking, fiber fracturing, and surface material loss presented in the failure ring. When the impact angle increased from 0° to 15°, the material loss area increased obviously in the fiber-oriented PE condition, except for the damage area tilt of the failure ring. When the PE condition increased from 15° to 30°, the damage was reduced, with only the matrix cracking and a small part of the fiber cracking; no material stripping occurred. When the PE was increased from 30° to 45°, the damage was further weakened, and only resin removal occurred. At an impingement angle of 45°, the resin removal area produced by the PA was larger than that by the PE. The above results show that the impact damage of a single jet on CFRP laminates does not show a simple decreasing trend with an increase in the impact angle, but it is related to the impact energy and damage mode. The material loss caused by a 15° oblique impact at 600 m/s may be greater than that caused by a normal impact.

**Figure 6:**
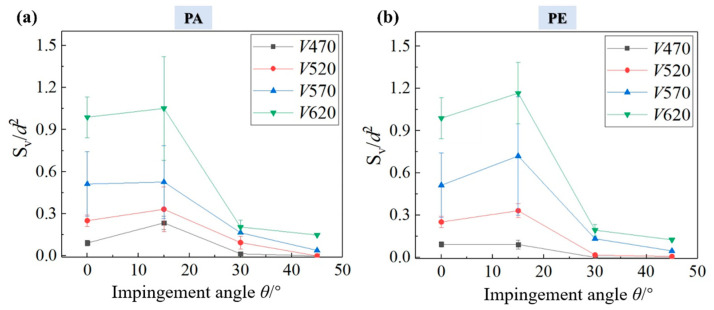
Comparison of dimensionless values of the surface visible damage area *S*_v_ at different velocities and impact angles for fiber orientations (**a**) PA and (**b**) PE.

As shown in Figure 6, the comparison of damage areas under different speeds and impact directions is presented, where the surface visible damage *S*_v_ on the vertical axis is dimensionless (divided by *d*^2^) to eliminate the influence of the jet diameter on the damage analysis. The results show that in most cases, the damage caused by a 15° oblique impact is slightly larger than that of a normal impact, while the increase in amplitude varies with different impact speeds. The damage areas from the 30° and 45° impacts are significantly smaller than that from a normal impact. In the case of a PA fiber orientation, the damage difference between a 15° oblique impact and a 0° impact gradually decreases as the impact speed increases, as shown in Figure 6a, while the results are the opposite in the case of a PE fiber orientation, as shown in Figure 6b, which show little difference in the damage area between 15° and 0° at a lower speed, while the damage area at 15° is significantly larger than that of a 0° direct impact at a higher impact speed.

**Figure 7:**
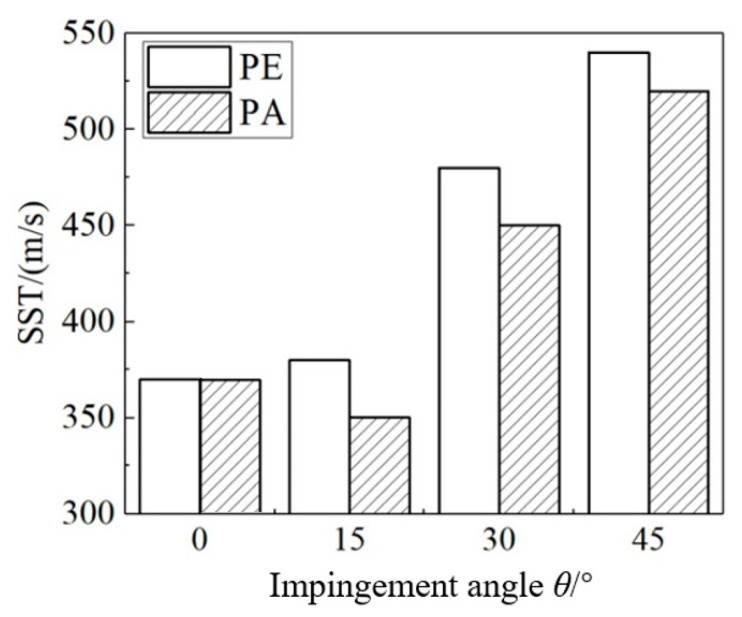
Comparison of the single-velocity threshold (SST) of samples with different impact directions under the impact of a single jet with a diameter of 4.5 mm.

Figure 7 shows the statistics of the single-shot velocity threshold (SST) of the CFRP specimens under a single-jet impact with an average diameter of 4.5 mm at seven different impact directions. The following patterns are revealed: (1) The SST generally increases as the impact angle increases, although this increase is not linear. The SST at 30° and 45° is significantly higher than that at 0°, while the SST at 15° shows little difference with that at 0°. (2) At the same angle, the SST of the PA orientation is lower than that of the PE orientation, indicating that the PA orientation is more prone to initial damage. In particular, the SST of the 15°-PA condition is even lower than that of the 0° condition.

#### 3.2.2. Effect of Fiber Orientation

**Figure 8:**
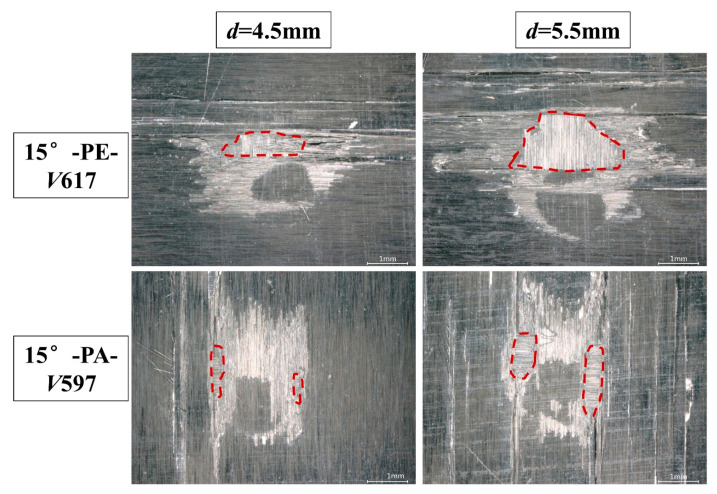
Damage comparison between the 15°-PE and 15°-PA conditions under two jet diameters with an impact velocity of 600 m/s. The areas circled by the dotted lines represent the area of material peeling (Sp).

Figure 8 shows the damage comparison of the 15°-PE and 15°-PA conditions under two jet diameters at a speed of approximately 600 m/s. It can be seen that the increase in jet diameter does not result in new damage patterns but deepens the existing matrix cracking and expands the surface delamination area. The difference in fiber orientation between PE and PA is also verified; that is, the fiber orientation of PE is more prone to large-area delamination as well as mass material loss in the impact projection direction, while the delamination damage for PA usually occurs on both sides of the failure ring and has a smaller area.

**Figure 9:**
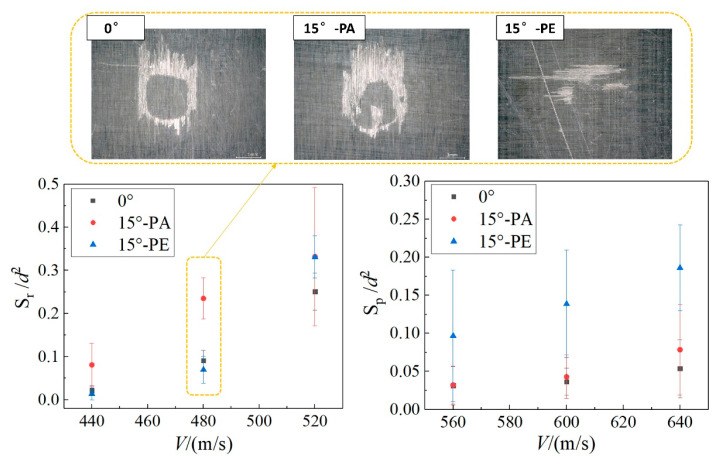
Comparison of the resin removal area Sr and surface peeling area *S*_p_ at impact conditions of 0°, 15°-PE, and 15°-PA.

According to Section 3.2.1, the damage extents caused by 30° and 45° oblique impacts are obviously small within the studied velocity range; therefore, only the three typical conditions of 0°, 15°-PE, and 15°-PA are analyzed in the following section. Figure 9 presents the resin removal area Sr and the top-ply peeling area *S_p_* under the three conditions of 0°, 15°-PE, and 15°-PA. Below a jet velocity of 500 m/s, the typical damage mode on the sample surface is resin removal, of which the area under the 15°-PA condition is significantly larger than that of the other two. Combined with the result that the SST velocity threshold of the 15°-PA condition in Figure 7 is also lower than that of the other two, the CFRP laminate impacted by the waterjet with the 15°-PA condition is more prone to resin removal damage. When the jet velocity increases to above 560 m/s, the damage mode of top-ply peeling and exposure of the second layer begins to appear on the surface. It is found that the 15°-PE condition is more likely to cause large-area peeling damage compared with the other two conditions due to the low transverse tensile strength of the laminate. Generally, a 15° oblique impact is more likely to cause more damage on the CFRP laminate than a normal impact by single waterjets. The PA fiber orientation is more likely to cause initial resin removal damage, while the PE fiber orientation can cause large-area peeling of the surface layer material.

**Figure 10:**
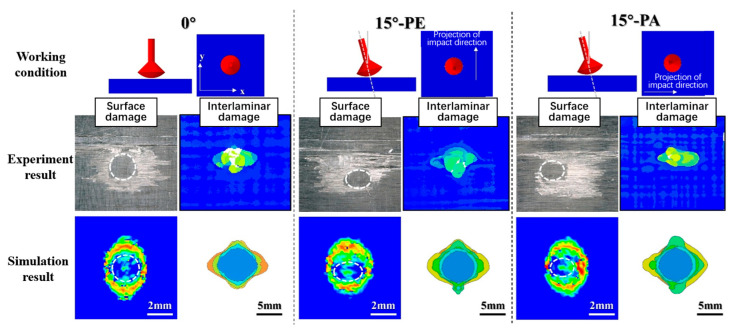
Comparison of experimental and simulation damage results from the impact of a waterjet with a diameter of 4.8 mm and a speed of 580 m/s under the three impact directions of 0°, 15°-PE, and 15°-PA.

The damage mechanism of an oblique impact was further revealed by the evidence provided by the simulation results of the mechanical response and damage evolution process, for which the simulation modeling can be found in Appendix A. Figure 10 shows the comparison of the experimental and simulation damage results from the impact of a waterjet under the three impact directions of 0°, 15°-PE, and 15°-PA. The damage results in the simulation were similar to those in the experiment both in shape and size, including the elliptical undamaged zone at the center under the oblique impact (marked as a white dotted circle), the asymmetric surface failure ring, and the internal delamination area, confirming the rationality of the simulation model in this paper.

**Figure 11:**
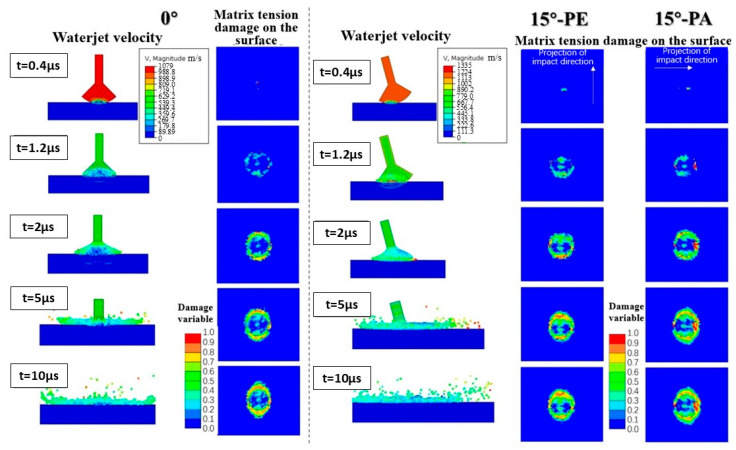
The variation in the waterjet morphology and the damage evolution of CFRP laminates with an impact speed of 580 m/s under the three impact directions of 0°, 15°-PE, and 15°-PA.

Figure 11 shows the evolution process of the surface matrix tensile damage (which has been proved in [20] to be the critical damage mode in a single-waterjet impact on a CFRP) under three working conditions. It can be clearly seen that the surface tensile failure ring shows a trend of uniform deepening in all directions, with symmetrical jet ejection of the lateral jet under the normal collision conditions. As for the 15° oblique impact, the surface tensile failure ring presents asymmetric damage patterns and deepens along the impact projection direction. According to the simulation results in Table 2, the maximum lateral jet velocity (1347 m/s) under the oblique impact of 15°-PE increases by about a quarter compared with the value under the normal impact condition (1079 m/s), and the occurrence time is obviously later than that under the positive impact condition. This phenomenon can be attributed to the acceleration of the lateral jetting under the superposition of the horizontal component of the impact velocity. The stress component under an oblique impact is also significantly different from that under a normal impact. The compressive stress component S_33_ along the thickness of the specimen is maximum for a normal impact, which is consistent with the reduction in the water-hammer pressure with an increase in the impact angle. On the other hand, S_23_ and S_13_, which represent the shear stress components, show their maximum value under 15°-PE and 15°-PA conditions, respectively. The strengthening of the lateral jet in the impact projection direction leads to an increase in the shear stress in the corresponding direction.

### 3.3. Discussion on the Damage Mechanism

**Figure 12:**
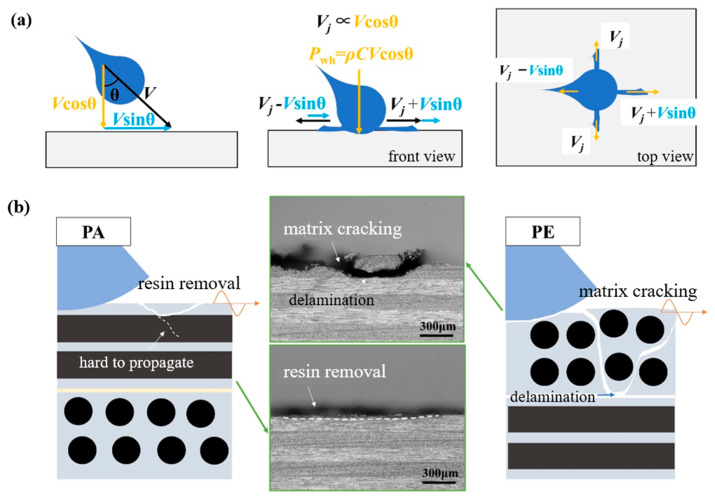
The internal mechanism of (**a**) oblique impact damage and (**b**) different damage modes caused by the fiber orientations PA and PE in a single-waterjet impact.

According to the above single-jet impact results, the following damage characteristics and corresponding mechanism analysis can be summarized:

1. An oblique impact at a small angle of 15° can lead to larger damage extents than a normal impact at 0° on CFRP laminates; a similar tendency was found in [16]. In an oblique impact, the velocity can be decomposed into a component perpendicular to the impact surface (Vcos θ) and a component parallel to the impact surface (Vsin θ). As shown in Figure 12, the vertical component Vcos θ dominates the magnitude of the water-hammer pressure, and the lateral jet velocity for a normal impact, Vj, can be considered proportional to Vcos θ, according to a previous investigation [12]. This can be written as Vj = *a*Vcos θ (where *a* denotes the proportional factor, which has been found to be 5~10 in previous studies [12,13]). Meanwhile, the lateral jet is enhanced along the projected impact direction, such that the jetting velocity can be regarded as Vj + Vsin θ, derived as V(*a*cos θ + sin θ). The shear force can be assumed to exhibit a positive correlation with the lateral jetting velocity. Notably, the jet velocity for an oblique impact along the projected direction may exceed that of the 0° normal impact (specifically, V(*a*cos θ + sin θ) > *a*V), generating greater shear forces and damage dimensions. When the value of *a* varies from 5 to 10, the critical oblique angle θ is 11~22°, which matches the observed phenomenon for the 15° oblique impact in the present study.
2. The PA fiber orientation is more prone to cause resin removal than PE, which results in a lower single-jet impact damage threshold velocity (SST) for PA than PE at the same impact angle. The damage difference caused by the fiber orientation is determined by the inherent anisotropy of the laminate. Since the reinforcing phase used in this study consists of unidirectional continuous fibers, the matrix–fiber interface in the PA direction is also continuous. When the lateral jet is spread along a PA fiber orientation, matrix microcracks are more likely to initiate and expand along the interface, leading to erosion and removal of the resin damage. Conversely, an impact along the PE direction can only influence the regions between fibers in the matrix, and the interface is discontinuous and not conducive to the expansion of matrix microcracks. Therefore, the PA fiber orientation is more likely to cause initial resin removal damage than PE, resulting in a lower damage threshold velocity for a single-waterjet impact. The damage mechanism of resin removal is also associated with the effect of surface roughness, which is further explained in [21] in terms of the relationship between resin removal and the fiber orientation.
3. A PE fiber orientation can lead to mass material peeling in comparison with PA, and the damage range is even much larger than from a normal impact. Due to the low transverse tensile strength of the laminate, the release tensile wave of the water-hammer pressure will generate matrix tensile cracks in this region and extend to the first interlaminar interface, which has been recounted in [6,22]. For the oblique impact shown in Figure 12, the velocity of the lateral jet as well as the induced shear force will be increased along the impact direction and cause delamination damage on the transverse region of the failure ring. The delamination region between layers overlaps with the matrix cracking and fiber breakage region within the surface failure ring, resulting in a larger area of material peeling than from the 0° normal impact. Conversely, if the same impact energy is exerted on CFRP laminates for a PA-orientation impact, the good longitudinal tensile performance of the laminate will inhibit the expansion of surface matrix cracks and thus block the intersection of interlaminar delamination and surface cracks, leading to minor material peeling along the impact direction.

### 3.4. Fitting Model of the Undamaged Central Area

**Figure 13:**
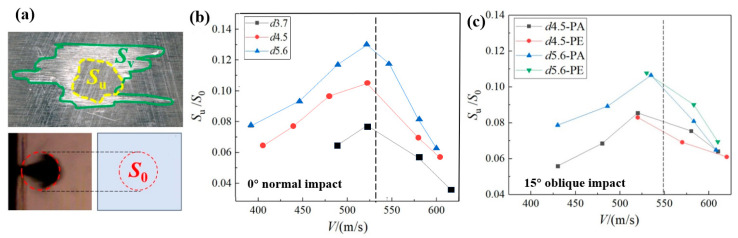
(**a**) Definition of the area parameters, and dimensionless results of the undamaged central area upon variations in the velocity and the diameter of the waterjet under (**b**) normal impact and (**c**) a 15° oblique impact.

The typical damage feature of the CFRP laminate after a single-jet impact is the surface failure ring, with the undamaged area defined as *S*_u_ in Figure 4. Previous studies [23,24,25] have shown that it is difficult to predict the undamaged center region of the ring by purely theoretical methods. Therefore, a fitting model is proposed in this section based on test data. According to Figure 13, an area parameter *S*_0_ based on the jet diameter is introduced for data dimensionless processing, which represents the area of a circle with jet diameter *d* as its diameter. The dimensionless results of *S*_u_/*S*_0_ in Figure 13b,c are presented in relation to the jet diameter *d*, the jet velocity *V*, and the impact angle *θ*, which can be described by

(1) $$\frac{S_{u}}{S_{0}}=f\left(V,d,\theta \right)$$

When the jet velocity is relatively low (in this case, lower than the corresponding values of the vertical dotted lines in Figure 13b,c), there is only a reasonable range of resin removal and minor matrix cracks. The roughly linear increasing trend of *S*_u_/*S*_0_ with increasing jet velocity conforms to the change trend predicted by the water-hammer-pressure contact theory. When the waterjet velocity continued to increase, obvious matrix cracking and fiber fractures began to appear and led to the unpredictable expansion of the failure ring and a decrease in *S*_u_ with a large dispersion. Therefore, the fitting model only considers cases that only cause surface resin removal. In addition, the influence of fiber orientation on *S*_u_/*S*_0_ can be ignored, so the model does not distinguish between the differences in fiber orientation.

According to Engel’s theory on the water-hammer pressure [24] and the position of the shock wave when it is about to exceed the extended boundary, the “contact radius” can be given by the following formula:

(2) $$R=\frac{d}{2}\frac{V}{C_{0}+2V}$$

in which *d* denotes the waterjet diameter, *V* denotes the waterjet velocity, and *C*_0_ is a constant that represents the acoustic velocity of the water.

In order to determine the relationship between the theoretical predicted contact radius and the undamaged range obtained by the test, the equivalent diameter *d*_u_ of the undamaged center of the sample is defined as follows:

(3) $$d_{u}=\sqrt{\frac{4S_{u}}{\pi }}$$

The release wave at the contact radius plays an important role in damage initiation at the failure ring, so it is assumed here that the “contact diameter” corresponding to the contact radius is a theoretical estimate of the equivalent diameter of the central undamaged region; that is,

(4) $${\left(\frac{d_{u}}{d}\right)}_{1}=\frac{V}{C_{0}+2V}$$

Another scholar, Wang [26], presented in their derivation of the contact radius that the wave causing tensile stress on the specimen surface should be a Rayleigh wave, so the influence of the Rayleigh wave velocity *C*_R_ should be taken into account when deducing the central undamaged region of the failure ring, and the formula given herein is used as another theoretical estimate of the equivalent diameter of the undamaged center:

(5) $${\left(\frac{d_{u}}{d}\right)}_{2}=\frac{V}{C_{R}}{\left[1+{\left(\frac{V}{C_{R}}\right)}^{2}\right]}^{-\frac{1}{2}}$$

**Figure 14:**
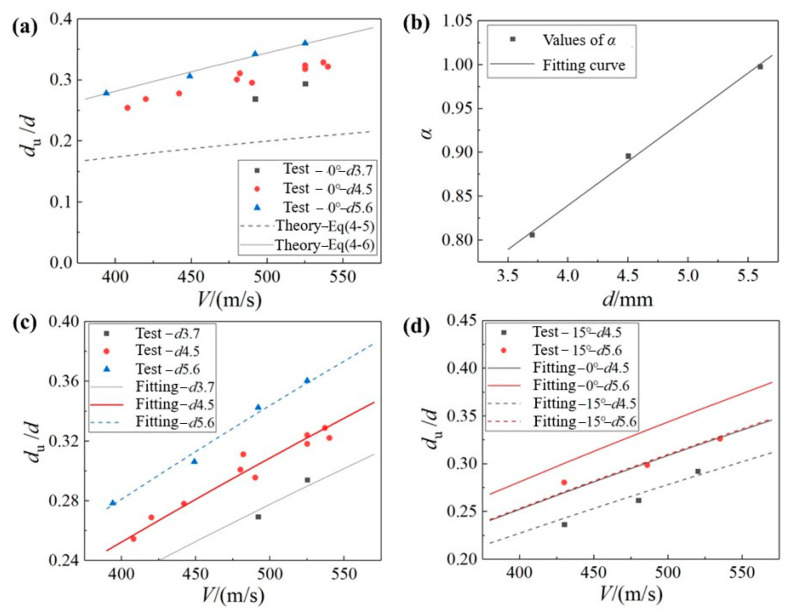
(**a**) Comparison of experimental values of *d*_u_/*d* (equivalent diameter ratio of the undamaged central region to the jet diameter) under 0° normal impact conditions at varying velocities and jet diameters versus two theoretical curves. (**b**) Fitting results of parameter *α* versus jet diameter *d*. (**c**) Experimental *d*_u_/*d* values and corresponding fitted curves for a 0° normal impact and (**d**) for a 15° oblique impact.

For the CFRP composite material in this paper, *C*_R_ = 1362 m/s. First, the 0° normal impact condition is considered. Figure 14a shows the test results for the ratio of the equivalent diameter of the central undamaged zone to the jet diameter *d*_u_/*d*, as well as the theoretical curves of Equations (4) and (5). According to the comparison results, Equation (4) underestimates the size of the central undamaged zone, which is consistent with the phenomenon previously noted in the literature and attributed to the intersecting paths of laterally ejected liquid particles at the contact boundary [12]. In contrast, Equation (5) demonstrates closer agreement with the experimental results. However, since this equation accounts solely for the jet velocity *V*, the parameters *α* and *β* are introduced to characterize the influence of the jet diameter and the impact angle, respectively, leading to the following modified damage estimation formula:

(6) $${\left(\frac{d_{u}}{d}\right)}_{\mathrm{fitting}}=\alpha \left(d\right)\cdot \beta \left(\theta \right)\cdot {\left(\frac{d_{u}}{d}\right)}_{2}=\alpha \left(d\right)\cdot \beta \left(\theta \right)\cdot \frac{V}{C_{R}}{\left[1+{\left(\frac{V}{C_{R}}\right)}^{2}\right]}^{-\frac{1}{2}}$$

First, the 0° normal impact condition is considered, and *β* is set to 1. The Levenberg–Marquardt (L-M) optimization algorithm [27] was adopted to fit the experimental points *d*_u_/*d* corresponding to the three jet diameters in Figure 14a, in accordance with Equation (6). The optimal solutions of parameter *α* were 0.806 (*d*3.7), 0.896 (*d*4.5), and 0.998 (*d*5.6), respectively. When the three fitted values are plotted on Figure 14b, they show that the parameter *α* basically satisfies a linear relationship with the jet diameter *d*, which is

(7) $$\alpha \left(d\right)=k\cdot d+b$$

in which the undetermined parameters *k* and *b* can be fitted by the data in Figure 14a. The results are *k* = 0.101 and *b* = 0.437. The estimated results of *α* in Equation (7) are brought into Equation (6) and presented in Figure 14c. It can be seen that the modified estimation equation can basically achieve good fitting results for the test values of *d*_u_/*d* (with a fitting-effect R-squared value of 0.943) for a normal impact.

Next, the effect of an oblique impact is considered. From the observation of the test data in Figure 13c, it is found that the undamaged center area of the oblique impact at the same diameter and speed will be smaller than that from a normal impact. This trend is consistent with the trend of the water-hammer pressure area decreasing with cos^2^ *θ*, so the function *β* describing the impact angle can be written as

(8) $$\beta \left(\theta \right)={\left(\cos\theta \right)}^{n}$$

in which *n* is the undetermined parameter. The L-M optimization algorithm was adopted to fit the *d*_u_/*d* values corresponding to the two jet diameters in Figure 13c at the 15° oblique impact test points. The optimal solutions for parameter *n* were 3.06 (*d*4.5) and 2.92 (*d*5.6), respectively. To simplify the model, parameter *n* was set as a constant (*n* = 3) and substituted into Equation (6) to obtain four fitted curves under the two jet diameters and two impact angles, as shown in Figure 14d. It can be seen that the model-fitting results, after considering the influence of the impact angle, are good (*R*^2^ = 0.932).

Substituting Equations (6)–(8) into Equation (1) for the *S*_u_ estimation yields:

(9) $$S_{u}=\frac{S_{u}}{S_{0}}S_{0}={\left(\frac{d_{u}}{d}\right)}^{2}\frac{\pi }{4}d^{2}=\frac{\pi }{4}d^{2}{\left(k\cdot d+b\right)}^{2}{\left(\cos\;\theta \right)}^{2n}\frac{V^{2}}{V^{2}+C_{R}^{2}}$$

To evaluate the predictive capability of the model established in this section, ten sets of experimental data not used in the parameter-fitting process of the aforementioned model were compared with the model predictions, as presented in Table 3. The results demonstrate that the model exhibits satisfactory predictive accuracy for the undamaged central region *S*_u_, with an average error within 10% and a maximum error of 15.4% across all estimates. Therefore, the theoretical model of single-jet impact damage developed in this section demonstrates robust predictive capabilities for the area of the undamaged central region within the surface failure ring dominated by resin removal.

## 4. Conclusions

This study conducted single-jet impact experiments on laminates with two fiber orientations (PE, PA) under multiple impact angles (0°, 15°, 30°, 45°). The damage characteristics of laminates under oblique impact conditions were systematically analyzed through experimental characterization and numerical simulations, revealing the critical role of impact orientation (including both the impact angle and the fiber orientation) in carbon fiber-reinforced polymer (CFRP) rain-erosion mechanisms. A theoretical model for waterjet impact damage was developed to quantitatively characterize the influence patterns of impact orientation. The principal findings are as follows:

1. The threshold velocity for single-jet-induced damage generally increases with the impact angle, while the damage severity decreases accordingly. However, a 15° oblique impact under high-velocity conditions may generate more extensive surface damage than a 0° normal impact. Finite-element modeling, validated by experimental data, suggests that this anomalous phenomenon stems from the increased lateral jet-particle velocity and resultant shear stress along the impact projection direction.
2. PA fiber orientations in the CFRP laminates exhibited lower threshold velocities and incubation periods compared with their PE counterparts at identical angles, whereas a PE orientation demonstrated greater susceptibility to large-area material delamination (particularly under a 15° high-velocity impact). This divergent behavior originates from differential matrix microcrack initiation and propagation along fiber–matrix interfaces, with PE interfaces showing reduced resistance to resin removal. The damage extent under an oblique impact in the PE orientation even exceeded that of normal impact scenarios.
3. Based on the theoretical formula for the water-hammer-pressure contact radius, the fitting model of the undamaged central area on the surface *S*_u_ after single-waterjet impact is established, which generally realizes the decoupling and quantitative description of the influences of jet velocity, jet diameter, and impact angle. This model demonstrates good predictive capability for matrix-dominated surface failure patterns, achieving maximum prediction errors of below 20% for the parameter of the undamaged surface center area across validation cases.
4. This study only analyzes the waterjet impact characteristics of unidirectional laminate composites without validating the universal applicability across other types of composites. Future research could focus on a broader spectrum of composites (including varying weaving patterns and matrix types), which would help to reveal the influence of material parameters on the waterjet impact resistance of composites.

## Figures and Tables

**Table 1:** Mechanical properties of T700/7901 unidirectional laminates.

*ρ*/(kg/m^3^)	*E*_11_/GPa	*E*_22_/GPa	*G*_12_/GPa	*V*_12_
1578	115	9	3.3	0.33
*X*_t_/MPa	*X*_c_/MPa	*Y*_t_/MPa	*Y*_c_/MPa	*S*_12_/MPa
2300	1050	42	143	116
Glass transition temperature (*T*_g_) of the resin/°C				110

**Table 2:** Simulation results of the waterjet velocity and maximum stress components under the three impact directions of 0°, 15°-PE, and 15°-PA.

Working Condition	0°	15°-PE	15°-PA
Maximum velocity of the lateral jetting *V*_j_ (occurrence time)	1079 m/s (1.8 μs)	1347 m/s (3.2 μs)	1293 m/s (2.8 μs)
Maximum value of the stress component S_33_ (occurrence time)	2.082 GPa (0.2 μs)	1.865 GPa (0.4 μs)	1.996 GPa (0.4 μs)
Maximum value of the stress component S_23_ (occurrence time)	0.485 GPa (1 μs)	0.501 GPa (1.2 μs)	0.453 GPa (0.8 μs)
Maximum value of the stress component S_13_ (occurrence time)	0.412 GPa (1 μs)	0.457 GPa (0.8 μs)	0.489 GPa (1.2 μs)

**Table 3:** Comparison of the prediction and test results of a single-waterjet impact.

*V* (m/s)	*d* (mm)	*θ* (°)	*S*_u_ (mm^2^)		
			Test Result	Prediction	Error
428	5.9	0	2.42	2.62	8.47%
471	6.1	0	3.33	3.46	4.10%
507	6.3	0	3.84	4.37	13.7%
514	5.9	0	3.29	3.64	10.5%
450	6.3	15	2.49	2.87	15.4%
535	6.1	15	3.21	3.52	9.80%
492	6.3	30	/	1.75	/
600	5.9	30	1.89	2.00	5.99%
557	6.1	45	/	0.58	/
610	5.9	45	/	0.61	/
Average error					9.71%
Maximum error					15.4%

**Figure A1:**
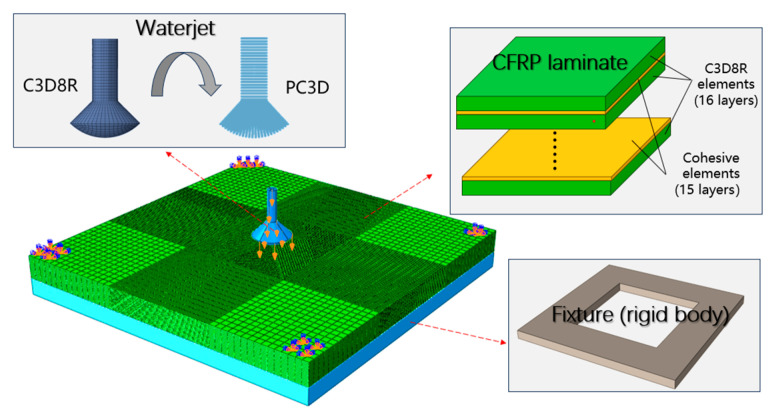
Finite-element model of a CFRP laminate impacted by a waterjet.

## Data Availability

The raw/processed data required to reproduce these findings cannot be shared at this time as the data also form part of an ongoing study. The raw data are available from the corresponding author upon reasonable request.

## Appendix A. Finite-Element Modeling

The simulation model can capture the damage process and stress distribution, which are difficult to measure in an experiment; hence, it is used in this paper to analyze the damage mechanism of an oblique impact. The whole numerical model is comprised of three components, as shown in Figure A1. The CFRP plate with a length and width of 30 mm is composed of 16 unidirectional plies stacked in the sequence of [0,90]_4s_, and each ply has one layer of elements, with a ply thickness of 0.15 mm. An 8-node reduced-integration brick element (C3D8R) is selected to mesh all the laminae. Cohesive elements (COH3D8) are inserted between neighboring plies to describe the behavior of the interlaminar interface (15 layers in total). After the mesh convergence study, the central local area of the plate is assigned to a refined mesh size (0.15 mm) for accurate calculations near the impacted region, while a coarser mesh (0.6 mm) is applied to the rest of the plate. Prediction of all the intralaminar damage behaviors is realized by subroutines of the constitutive models [28], and the interlaminar damage is predicted by the cohesive elements based on a bilinear traction–separation law [29], all of which have been presented and validated in our previous studies [20]. The back surface of the plate is mounted onto the rigid fixture with a hard contact, and some nodes near the edge of the front surface are fixed to constrain the through-thickness displacement of the plate.

The movement of a waterjet is calculated by the Smoothed Particle Hydrodynamics (SPH) formulation. Instead of a grid, SPH particles interact with each other following a kernel interpolation within a smoothing length, thus avoiding the occurrence of severe mesh distortion during a large deformation. The waterjet is modeled by solid elements (C3D8R) in advance and these “parent” elements are converted into SPH particles (PC3D) at the beginning of the analysis.

The Mie–Gruneisen Equation of State (EOS) is available for the description of the hydrodynamic behavior of water. Here, the linear *U*_s_–*U*_p_ Hugoniot form of the Mie–Gruneisen EOS is presented; for a detailed derivation, refer to [30].

(A1) $$p=\frac{\rho_{0}C_{0}^{2}\eta }{(1-s\eta {)}^{2}}\left(1-\frac{\Gamma_{0}\eta }{2}\right)+\Gamma_{0}\rho_{0}E_{m}$$

In the above equation, *p* is the Hugoniot pressure, *E*_m_ is the internal energy per unit mass, *Γ*_0_ is a Gruneisen material constant, and *ρ*_0_ is the reference density. η is the nominal volumetric compressive strain defined by $1-\frac{\rho_{0}}{\rho }$, in which *ρ* is the material density. $\rho_{0}C_{0}^{2}\eta$ is equivalent to the elastic bulk modulus at small nominal strains. *C*_0_ and *s* are introduced to define the linear relationship between the linear shock velocity *U*_s_ and the particle velocity *U*_p_ as follows:

(A2) $$U_{s}=C_{0}+sU_{p}$$

The values of the coefficients for water are referred to in [20,31], with the density *ρ*_0_ = 1000 kg/m^3^, the sound speed *C*_0_ = 1489 kg/m^3^, and the two material constants s = 1.79 and *Γ*_0_ = 1.65.
